# Multidimensional pain assessment of preterm newborns at the 1^st^, 3^rd^ and 7^th^ days of life

**DOI:** 10.1590/S1516-31802007000100006

**Published:** 2007-01-04

**Authors:** Ana Beatriz Mello Serpa, Ruth Guinsburg, Rita de Cássia Xavier Balda, Amélia Miyashiro Nunes dos Santos, Kelsy Catherina Nena Areco, Clóvis Araújo Peres

**Keywords:** Newborn infant, Premature infant, Pain, Pain measurement, Pain threshold, Recém-nascido, Prematuro, Dor, Avaliação da dor, Limiar da dor

## Abstract

**CONTEXT AND OBJECTIVE:**

It is challenge to assess and treat pain in premature infants. The objective of this study was to compare the multidimensional pain assessment of preterm neonates subjected to an acute pain stimulus at 24 hours, 72 hours and seven days of life.

**DESIGN AND SETTING:**

Prospective cohort study, at Universidade Federal de São Paulo.

**METHODS:**

Eleven neonates with gestational age less than 37 weeks that needed venepuncture for blood collection were studied. The exclusion criteria were Apgar score < 7 at five minutes, presence of any central nervous system abnormality, and discharge or death before seven days of life. Venepuncture was performed in the dorsum of the hand, and the heart rate, oxygen saturation and pain scales [Neonatal Facial Coding System (NFCS), Neonatal Infant Pain Scale (NIPS), and Premature Infant Pain Profile (PIPP)] were assessed at 24 hours, 72 hours and 7 days of life. NFCS and NIPS were evaluated prior to procedure (Tpre), during venepuncture (T0), and two (T2) and five (T5) minutes after needle withdrawal. Heart rate, O_2_ saturation and PIPP were measured at Tpre and T0. Mean values were compared by repeated-measurement analysis of variance.

**RESULTS:**

The pain parameters did not differ at 24 hours, 72 hours and 7 days of life: heart rate (p = 0.22), oxygen saturation (p = 0.69), NFCS (p = 0.40), NIPS (p = 0.32) and PIPP (p = 0.56).

**CONCLUSION:**

Homogeneous pain scores were observed following venepuncture in premature infants during their first week of life.

## RESUMO

**CONTEXTO E OBJETIVO:**

É um desafio avaliar e tratar a dor do bebê prematuro. O objetivo deste estudo foi comparar, diante de um mesmo estímulo doloroso agudo, as respostas multidimensionais à dor obtidas ao longo da primeira semana de vida de prematuros.

**TIPO DE ESTUDO E LOCAL:**

Coorte prospectiva, na Universidade Federal de São Paulo.

**MÉTODOS:**

Estudo de 11 neonatos com idade gestacional inferior a 37 semanas e necessidade de punção venosa para coleta de sangue, sendo excluídos aqueles com Apgar < 7 aos cinco minutos, alterações do sistema nervoso central e os que faleceram ou tiveram alta até sete dias de vida. A punção venosa foi feita no dorso da mão e avaliou-se a freqüência cardíaca, a saturação de oxigênio e as seguintes escalas de dor: NFCS (Neonatal Facial Coding System), NIPS (Neonatal Infant Pain Scale) e PIPP (Premature Infant Pain Profile) com 24, 72 horas e no sétimo dia de vida. A NFCS e a NIPS foram pontuadas antes da punção venosa (Tpré), durante (T0), dois (T2) e cinco (T5) minutos após. A freqüência cardíaca, a saturação de oxigênio e a PIPP foram analisadas em Tpré e T0. Compararam-se os valores médios das variáveis nos três momentos por análise de variância com medidas repetidas.

**RESULTADOS:**

Não houve diferenças no primeiro, terceiro e sétimo dias para freqüência cardíaca (p = 0,22), saturação de oxigênio (p = 0,69), NFCS (p = 0,40), NIPS (p = 0,32) e PIPP (p = 0,56).

**CONCLUSÃO:**

Houve homogeneidade da avaliação da dor causada por punção venosa em prematuros, ao longo da primeira semana de vida.

## INTRODUCTION

Diagnostic and/or therapeutic procedures are required for the survival of critically ill newborn babies, and the possible pain generated by such procedures has been a source of concern. Simons et al.^1^ studied 151 neonates during their first 14 days of neonatal intensive care unit (NICU) stay and found that each of them was subjected to an average of 14 painful procedures per day. Prestes et al.^2^ evaluated four university neonatal units in São Paulo for one month in 2001 and found that an average of three to five potentially painful procedures were performed every day.

Pain relief measures are hardly ever used for neonates that are subjected to such potentially painful procedures. Specific analgesic or anesthetic treatment is utilized for only 3% of newborn infants undergoing painful procedures, while nonspecific analgesia is given to 30% of them for reasons other than the procedures *per se*.^3^ According to Simons et al.,^1^ 15-32% of 1,375 patients-day in NICU during their first two weeks of life received some dose of analgesia every day. These data are concordant with those of Prestes et al.,^2^ who found that, out of 1,025 patients-day in NICU, 23% received at least one dose of analgesics.

The most frequently cited reasons for pain undertreatment are the various myths surrounding painful experiences in neonatal populations: the likely incapacity of newborn infants to feel and express their pain, the difficulty in measuring the painful phenomenon in pre-verbal infants and the insufficient availability of effective and safe therapeutic options for pain treatment. However, among these reasons, the gap between scientific knowledge and practical management is mainly due to the difficulty of pain assessment in pre-verbal infants.^4^ Pain evaluation in neonatal populations is not an easy task: the subjective nature of the painful experience and the existence of few reliable and valid evaluation instruments with clinic applicability, for measuring pain presence and intensity are difficult barriers to surmount.^5-7^ Moreover, especially with regard to premature infants at different phases of their central nervous system growth and development, the responses to repetitive pain may change over time, thereby making evaluation and treatment of pain very difficult.^8^

## OBJECTIVE

The objectives of this study were to analyze pain expression among preterm newborn infants following repetitive pain stimuli and to investigate whether physiological and/or behavioral pain is amplified or habituated during the first week of life.

## METHODS

This prospective study included preterm newborn infants admitted to the NICU of São Paulo Hospital, Universidade Federal de São Paulo — Escola Paulista de Medicina, from January to October 2003, following approval by the Research Ethics Committee of Universidade Federal de São Paulo. Written consent from the mother was required before patients were included in the study.

These premature infants included had gestational ages greater than or equal to 24 weeks and less than 37 weeks,^9^ presented adequate weight for the gestational age^10^ and had a postnatal age greater than or equal to 24 hours. For all of them, there was a need for venous puncture to collect blood for laboratory tests. The indication for laboratory tests was assessed by an attending neonatologist who was unrelated to the study.

Patients with conditions that might potentially alter the response to painful procedures were excluded, namely: five-minute Apgar score of less than 7; chromosomal disorders or central nervous system malformations; grade III and/or grade IV intraventricular hemorrhage;^11^ and drug abuse by the mother, including reported use of alcohol, marijuana, cocaine, cocaine byproducts and opioids. Furthermore, neonates who were discharged from hospital or who died within seven days of life were excluded.

The painful acute stimulus evaluated was venous puncture for blood collection. After cleansing the area with alcoholic chlorhexidine, the puncture was performed by means of a 25/27 gauge needle in the dorsum of the newborn's hand by the neonatologist or by the nurse in charge of the neonate. Only the first attempt at blood collection was considered for the study.

Venous puncture was performed at 24 hours of life, at around 72 hours of life and then on the seventh day of life. The newborns were assessed during the procedure, at the following times:

- **Tpre**: prior to performing the painful stimulus, after the newborn had been at rest for at least 30 minutes, without receiving any type of handling.
- **T0**: immediately after introducing the needle.
- **T2:** two minutes after needle removal.
- **T5:** five minutes after needle removal.

The following tools were used for evaluating the newborn's pain, by the same observer for all patients:

- Heart rate and oxygen saturation were evaluated using a pulse oximeter with the sensor placed on the foot 30 minutes before **Tpre**. Evaluation of these physiological pain parameters was carried out on the first, third and seventh days of life.
- Neonatal Facial Coding System (NFCS)^12^ at **Tpre**, **T0**, **T2** and **T5**, on the first, third and seventh days of life.
- Neonatal Infant Pain Scale (NIPS)^13^ at **Tpre**, **T0**, **T2** and **T5**, on the first, third and seventh days of life.
- Premature Infant Pain Profile (PIPP)^14^ at **Tpre** and **T0**, on the first, third and seventh days of life.
- Photograph of the infant's face taken using a digital camera without flash at **T0**, i.e. just after introducing the needle into the newborn's skin, on the first and third days of life. These photographs were taken by a second observer who was not assessing the pain scales and who was also in charge of the chronometer for controlling the times at which the evaluations had to be done. The pictures were scored according to NFCS^15^ criteria. The scores were attributed at the end of the data collection, with the photographs randomly arranged, by three observers who had been trained to recognize facial pain features and who were unaware of the scores obtained at the bedside.

With regard to the power of the study, it was determined that the pain evaluation measurements should be repeated on the same newborn infants on three different occasions (first, third and seventh days of life). It was expected that, between the first and seventh days of life, there would be a two-point difference in NFCS scores during venous puncture, with a 1.5-point standard deviation. Hence, in order to have a 20% beta error and a 5% unidirectional alpha error, it would be necessary to study 14 neonates.^16^

Comparisons between the pain scales on the first, third and seventh days of life were made by repeated-measurement analysis of variance (RM-ANOVA), taking into consideration spherical measurements. For NFCS and NIPS, the summary measurement analyzed was the area below the curve formed by the mean results obtained at **Tpre**, **T0**, **T2** and **T5**, for each of the three days evaluated.^17^ To assess the agreement between the three observers regarding photograph scoring, intraclass correlation coefficients were analyzed^18^ and classified as good, regular and bad.^19^ Statistical analysis was performed using the Statistical Package for the Social Sciences (SPSS), version 12.0, and p ≤ 0.05 was considered significant.

## RESULTS

From January to October 2003, 47 neonates of gestational age less than 37 weeks were admitted to the NICU. After excluding patients discharged from hospital before the seventh day of life, deaths, those with five-minute Apgar scores of less than seven, those with chromosomal disorders or central nervous system malformations, and patients presenting grades III and/or IV intra-ventricular hemorrhage, it was possible to include 14 newborns in the study. However, three mothers refused consent. Hence, the population of this study comprised 11 premature infants.

With regard to demographic data, the patients’ mean gestational age was 30 weeks, ranging from 27 to 36 weeks, and seven of them were less than 32 weeks old. The average weight at birth was 1,615 g, and the range was from 1,070 to 3,375 g. Five newborns weighed less than 1,500 g at birth. Four were male and seven were female. Among these 11 patients, eight (73%) were born by means of cesarean section, and all the mothers received locoregional anesthesia at delivery. The five-minute Apgar score was nine for all these 11 neonates.

With regard to neonatal morbidity, four babies presented respiratory distress syndrome, two of them transient tachypnea, four of them early-onset sepsis and four of them hypoglycemia. The main devices and medications utilized for the babies in the study are shown in Table 1. It is important to note that four patients were receiving continuous fentanyl infusion during the first pain evaluation, at 24 hours of life, and this also occurred with one neonate at the time of the second evaluation (72 hours of life) and with two patients at the seventh day of life.

**Table 1.** Number (percentage) of newborn infants with devices and medication for vital support on the first, third and seventh days of life

	1^st^ day	3^rd^ day	7^th^ day
**Incubator**	10 (91%)	10 (91%)	10 (91%)
**Venous access**	11 (100%)	10 (91%)	9 (82%)
**Mechanical ventilation by tracheal tube**	6 (54%)	2 (18%)	2 (18%)
**Nasal continuous distending pressure**	1 (9%)	2 (18%)	2 (18%)
**Vasopressors**	Zero	1 (9%)	2 (18%)
**Antibiotics**	4 (36%)	4 (36%)	6 (54%)
**Surfactant**	6 (54%)	zero	zero
**Fentanyl**	4 (36%)	1 (9%)	2 (18%)

The neonates were subjected to an average of 11 potentially painful procedures during their first 24 hours of life, 24 procedures du ring their first 72 hours of life and 36 procedures during their first week of life, counting only arterial, venous, capillary and liquor punctures and tracheal tube insertions (Table 2).

**Table 2.** Median number (minimum – maximum) of painful procedures performed from birth to 24 hours of life, to 72 hours of life and up to the seventh day of life of newborn infants

	Birth to 24 hours	Birth to 72 hours	Birth to 7 days
**Heel stick**	7 (2-12)	13 (4-21)	22 (10-46)
**Venous puncture**	2 (1-6)	6 (1-9)	6 (3-13)
**Arterial puncture**	2 (0-7)	4 (0-12)	7 (2-19)
**Lumbar puncture**	0 (0-4)	0 (0-5)	0 (0-5)
**Endotracheal intubation**	0 (0-1)	1 (0-2)	1 (0-3)

With regard to physiological response to pain (Table 3), there was a slightly higher elevation of the heart rate in response to introducing a needle into the back of the patients’ hands on the third and seventh days of life, in comparison with the smaller elevation noticed on the first day of life. However, there was no statistical difference in heart rate, between measurements made before and during the painful stimulus, for any of the three occasions evaluated (RM-ANOVA; p = 0.22). The oxygen saturation decreased by about 2% of its basal value during the painful stimulus at all three of the observation times, i.e. there was no statistical difference in the mean oxygen saturation, between measurements made before and during the painful stimulus, at 24 and 72 hours and at seven days of life (RMANOVA: p = 0.69).

**Table 3.** Heart rate (beats per minute) and oxygen saturation (%) prior to (Tpre) and during (T0) the painful stimulus, on the first, third and seventh days of life of newborn infants

	1^st^ day of life	3^rd^ day of life	7^th^ day of life
*Heart rate (bpm)*			
Tpre	134 ± 12	134 ± 13	137 ± 10
T0	136 ± 15	141 ± 12	146 ± 7
*O_2_ saturation (%)*			
Tpre	96 ± 3	96 ± 4	95 ± 2
T0	94 ± 5	94 ± 2	93 ± 2

*Repeated-measurement analysis of variance (RM-ANOVA): 1^st^ day = 3^rd^ day = 7^th^ day (p = 0.22 for heart rate and p = 0.69 for O_2_ saturation).*

Pain evaluation using the NFCS and NIPS scales showed that there was a slight increase in T0 scores (at the moment of the painful stimulus), with a return to basal values five minutes after venous puncture, on the first, third and seventh days of life (Table 4). The PIPP scores were low on all three observation days (Table 4). Statistical analysis showed that the measurements presented sphericity (a constant relationship between the measurements on all three observation days), without significant differences between the values obtained at 24 hours, 72 hours and seven days of life for NFCS (p = 0.40), NIPS (p = 0.32) and PIPP (p = 0.56).

**Table 4.** Neonatal Facial Coding System (NFCS) and Neonatal Infant Pain Scale (NIPS) scores prior to (Tpre), during (T0) and two (T2) and five (T5) minutes after the painful stimulus, and Premature Infant Pain Profile (PIPP) score on the first, third and seventh days of life

	1^st^ day of life	3^rd^ day of life	7^th^ day of life
*NFCS*			
Tpre	0.4 ± 1.3	0.3 ± 1.0	Zero
T0	3.6 ± 4.0	2.5 ± 3.0	3.0 ± 3.7
T2	1.5 ± 2.7	0.7 ± 2.1	1.7 ± 3.2
T5	0.5 ± 1.3	zero	0.2 ± 0.6
*NIPS*			
Tpre	0.3 ± 1.3	0.1 ± 0.3	Zero
T0	1.9 ± 1.5	1.1 ± 1.5	1.7 ± 1.3
T2	1.0 ± 1.6	0.4 ± 1.3	0.8 ± 1.6
T5	0.2 ± 0.8	zero	0.2 ± 0.6
*PIPP*	1.1 ± 1.8	1.2 ± 1.5	1.3 ± 1.5

*Repeated-measurement analysis of variance (RM-ANOVA): 1^st^ day = 3^rd^ day = 7^th^ day (NFCS p = 0.40; NIPS p = 0.32; PIPP p = 0.56).*

Finally, in the evaluation of facial movement responses to the painful stimulus, using photographs taken at the moment of venous puncture on the first and third days of life, there was good agreement between the three observers regarding the photos from the first day of life (intraclass correlation coefficient 0.80; 95% CI 0.61-0.92) and the third day of life (intraclass correlation coefficient 0.92; 95% CI 0.84-0.97). Taking the mean NFCS score from the three observers for each baby photographed as the final pain score for each patient, the means for the first and third days of life were compared and no statistical difference was found between them (first day of life 4.5 ± 1.7 and third day of life 4.2 ± 2.2, using paired Student t test: p = 0.79).

## DISCUSSION

There is a gap in the existing knowledge regarding the presence and consequences of pain and its application in clinical practice.^20^ To overcome this gap in relation to neonates, there are at least two core points to be addressed. The first is the difficulty in evaluating pain over this age range, while such evaluation is essential for instituting adequate treatment.^6^ The second relates to the paucity of safe, effective and well-studied therapeutic analgesic options for critically ill newborn infants.^21^

The present study relates to questions inherent to pain evaluation during the neonatal period. Clinical interpretation of newborns’ response to nociceptive stimulus is complicated because of the limited sensitivity of the tools available. Moreover, prematurity, postnatal age, coexistent diseases and the sensitivity of the healthcare professionals who need to interpret the pain measurement tools applied to newborn infants further complicate pain evaluation among patients of this age.^22^

There is evidence in the literature that preterm infants subjected to a great number of painful procedures show physiological pain response exacerbation and facial movement attenuation during subsequent acute painful stimulus.^23^ Exposure to repetitive painful stimuli may also attenuate the overall pain response, as assessed on a multidimensional scale such as the Premature Infant Pain Profile (PIPP), and such attenuation is modulated by the severity of the clinical status.^24^ In a study carried out on 136 infants who were subjected to capillary puncture for blood collection at a corrected gestational age of 32 weeks, the number of previous invasive procedures and the gestational age at birth were associated with the attenuation of facial movement response to painful stimulus and with increased sympathetic cardiac activity.^25^ Another study analyzed 87 premature infants at a corrected gestational age of 32 weeks who were subjected to capillary puncture and showed that for those born before 28 weeks, but not for those born at 29 to 32 weeks, there was a correlation between the number of previous painful procedures and the attenuation of facial movement response to painful stimulus (but not the autonomic response), regardless of primary disease severity and morphine use.^26^

On the other hand, a study on infants born at full term showed that repeated exposure to acute painful procedures increased the facial movements and the duration of crying during capillary puncture, and also increased the score attributed by adults to babies at the moment of puncture.^27^ Likewise, premature infants evaluated at 32 weeks of corrected gestational age showed increased facial and body movements and heart rate elevation in response to handling, when subjected to painful stimulus prior to such handling. The more premature the infants are, the less able they are to perform effective stress sign self-regulation.^28^

In the light of these often-conflicting data, the question of how to evaluate pain among premature infants who cannot verbalize pain and are subjected to multiple painful procedures required for their survival still persists. This pilot study evaluated pain expression in preterm infants during a small window of the hospitalization period: the first week of life, when painful procedures are more frequently needed and vital support measures are more necessary.^1,29^

The patients chosen for this study were those hospitalized in neonatal intensive care units: premature infants at several gestational ages who were suffering from various clinical problems. If, on the one hand, such diversity enhances the possibility of generalizing the findings, it is on the other hand a limitation by not focusing on preterm infants with more homogenous cha racteristics who would thus show the presence or absence of better-defined factors that might interfere in neonatal pain expression. It must be noted that the present study had low sampling power, thereby making its results more important for planning future research on this topic than for immediate application in terms of pain evaluation in neonatal intensive care.

The use of intravenous analgesia was not an exclusion factor, since the objective of the work was to verify the longitudinal evolution of pain response over the first seven days of life of premature infants, including patients who were seriously ill, under mechanical ventilation and subjected to multiple painful procedures, i.e. situations in which the use of opioid analgesia in the NICU is indicated. Opioid administration in critically ill babies may reduce the effects of exposure to repetitive pain and attenuate bio-behavioral pain response,^25^ but this issue is controversial.^26^ On the other hand, recent evidence has indicated that the use of morphine in ventilated preterm infants, initiated within the first four hours of life, may reduce the number of survivors without serious intracranial hemorrhage and/or periventricular leukomalacia.^21^

The patients studied here suffered an average of 11 procedures over the first 24 hours of life and, by the age of one week, they had been subjected to 36 procedures, only counting arterial, venous, capillary and liquor punctures and tracheal tube insertions. These data are concordant with the findings of Simons et al.^1^ over the first 14 days of life, who observed that their patients were subjected to 14 painful procedures per day, and those of Prestes et al.,^2^ who found that three to five painful procedures per day were applied to babies of various postnatal ages hospitalized in Brazilian intensive care units. As already discussed, this exposure to repetitive painful stimuli may attenuate bio-behavioral response to pain.^8,23,25,26,30^

The data presented in the present study, however, did not show any modifications in the physiological response to pain (heart rate and oxygen saturation), on scales validated for pain evaluation at the bedside (NFCS, NIPS and PIPP), or in evaluations of facial movement response to painful stimulus through photographic images taken during this stimulus, during the first week of life. It is important to emphasize that the tools utilized in the present study had been validated for evaluating acute pain during the neonatal period, at the bedside.^5,7^ Moreover, the excellent agreement between the different observers regarding the application of the NFCS to the photographs taken during venous puncture in the dorsum of the hand at 24 and 72 hours of life can be highlighted.

In view of these findings, and taking into account the limitation of the sampling power, some hypotheses may be formulated to explain them. First, the studies that have observed attenuation of the behavioral response to pain after repetitive nociceptive stimulus were generally on newborn infants at 32 weeks of postconception age and at around three or four weeks of postnatal age.^23-26,28^ It was not known, however, whether such attenuation could occur as early as the end of the first week. The results from the present study indicate that there is no modification in the multidimensional response to pain during the first seven days of life. Furthermore, according to Grunau et al.,^26^ bio-behavioral adaptation after repeated exposure to acute painful procedures seems to be caused by modifications in the regulation of the hypothalamus-hypophysis-adrenal axis, which occurs in patients who are at a more immature neurological development stage. In the present study, only three newborn infants had a gestational age of less than or equal to 28 weeks, while four were between 29 and 32 weeks and four were between 33 and 36 weeks.

Finally, another point that can be raised to explain the results presented is the fact that the painful stimulus comprised blood collection through venous puncture in the back of the hand, whereas most of the pre vious studies used capillary puncture.^23,25,26,30^ It is known that venous puncture in the back of the hand is less painful and more effective than capillary puncture for blood collection during the neonatal period.^31,32^ Hence, this could explain the low pain scale scores observed during the painful procedure and, perhaps, the lack of modification in behavioral response to venous puncture after exposure to multiple painful procedures during the first week of life.

## CONCLUSIONS

The response to pain triggered by venous puncture in the back of the hand was homogeneous on the first, third and seventh day of life, among newborn infants of 27 to 36 weeks of gestational age who were hospitalized in the NICU and exposed to an average of 12 painful procedures over the first 24 hours of life and 36 procedures during the first week of life. That is, the tools utilized for acute pain evaluation showed consistent results over the first week of life of preterm patients. There is a need, however, to expand this work to subsequent weeks of life, with stratification of the pain response according to patients’ neurological immaturity, the severity of the disease and the number of previous painful procedures.
